# Cell-Specific *PEAR1* Methylation Studies Reveal a Locus that Coordinates Expression of Multiple Genes

**DOI:** 10.3390/ijms19041069

**Published:** 2018-04-03

**Authors:** Benedetta Izzi, Fabrizia Noro, Katrien Cludts, Kathleen Freson, Marc F. Hoylaerts

**Affiliations:** 1 Department of Cardiovascular Sciences, Center for Molecular and Vascular Biology, University of Leuven, 3000 Leuven, Belgium; Katrien.cludts@uzleuven.be (K.C.); Kathleen.freson@Kuleuven.be (K.F.); Marc.hoylaerts@kuleuven.be (M.F.H.); 2 Department of Epidemiology and Prevention, IRCCS Istituto Neurologico Mediterraneo Neuromed, Via dell’Elettronica, 86077 Pozzilli (IS), Italy; fabrizia.noro@moli-sani.org

**Keywords:** *PEAR1*, DNA methylation, chromosome interactions

## Abstract

Chromosomal interactions connect distant enhancers and promoters on the same chromosome, activating or repressing gene expression. *PEAR1* encodes the Platelet-Endothelial Aggregation Receptor 1, a contact receptor involved in platelet function and megakaryocyte and endothelial cell proliferation. *PEAR1* expression during megakaryocyte differentiation is controlled by DNA methylation at its first CpG island. We identified a *PEAR1* cell-specific methylation sensitive region in endothelial cells and megakaryocytes that showed strong chromosomal interactions with *ISGL20L2*, *RRNAD1*, *MRLP24*, *HDGF* and *PRCC*, using available promoter capture Hi-C datasets. These genes are involved in ribosome processing, protein synthesis, cell cycle and cell proliferation. We next studied the methylation and expression profile of these five genes in Human Umbilical Vein Endothelial Cells (HUVECs) and megakaryocyte precursors. While cell-specific *PEAR1* methylation corresponded to variability in expression for four out of five genes, no methylation change was observed in their promoter regions across cell types. Our data suggest that *PEAR1* cell-type specific methylation changes may control long distance interactions with other genes. Further studies are needed to show whether such interaction data might be relevant for the genome-wide association data that showed a role for non-coding *PEAR1* variants in the same region and platelet function, platelet count and cardiovascular risk.

## 1. Introduction

The identification of long-range interactions between chromosome regions, separated by more than 100,000 bases led to the discovery of intra-chromosomal loops that juxtapose downstream enhancers close to several promoter regions. These chromosomal interactions play a key role in gene expression control [1], as they can often allow or impede interactions between the promoters and their closed target genes in the intervening DNA sequence [2,3,4,5,6,7,8,9,10,11]. Sequence-specific DNA-binding proteins may guide this process by directly repositioning these loci to relevant chromatin compartments [12,13,14,15,16]. These architectural proteins (i.e., CTCF, cohesin, etc.) are genome-wide bound to promoter-genome interactions and may interfere with gene expression through several mechanisms [17,18,19,20,21] including those that depend on DNA methylation and, moreover, such long-distance control can be coordinated in a cell-specific manner. DNA methylation at promoters and CpG islands is traditionally known to inhibit target gene expression by regulating the binding of transcription modulators to the promoter [22,23]. However, more and more examples of DNA methylation-dependent increases in gene expression are being described [24,25,26,27]. Of note, long-range interactions between CpGs and target genes have been reported [28,29]. Recently, the function of trans or long-range actions of CpG methylation has been revealed by a genome-wide study, performed on various cancer types. In this analysis, the correlation between DNA methylation at distal regulatory regions and long-range target gene expression was shown to be significantly stronger than the correlation with the nearby promoter methylation [30]. 

Recently, also thanks to the effort of several genomic consortia (BLUEPRINT [31,32], ROADMAP, ENCODE [33], The International Human Epigenome Consortium (IHEC) [34]) promoter capture Hi-C was used to unravel such interactions and this has been applied with success to interpret several of the non-coding disease- and trait-associated variants [35,36,37,38,39,40]. By using maps of long-range loops between enhancers and promoters it was possible to identify genes that are regulated by a disease-associated noncoding variant. These variants are in fact often located in regions at a considerable distance from an annotated gene or are in the close proximity of DNAse I hypersensitive loci [41], suggesting they might regulate gene transcription by altering the function of distal regulatory elements. This is also the case of long-range contacts in human blood cell types, where over 2500 potential disease genes were combined with a database of distal disease-associated variants [41].

*PEAR1* encodes for the Platelet-Endothelial Aggregation Receptor 1, a contact receptor particularly expressed in platelets, megakaryocytes and endothelial cells [42,43,44]. Many genetic studies, including GWAS studies, have reported *PEAR1* common genetic variants to be important for both platelet and endothelial cell function variability [45,46,47,48,49,50,51,52]. Interestingly, particular attention has been devoted to rs12041331, a variant located in the first intron of the gene and known to be coupled to *PEAR1* DNA methylation changes [53]. This variant has been associated with variability of platelet aggregation (before and after anti-platelet therapy) [48,51,52], cardiovascular outcomes [46], PLT and MPV [54]. Beyond this studies, more recent attempts looking for new and more rare variants in the region [47] or taking advantage of larger sample size and exome Chip coverage [54,55], both failed in identifying any coding missense or nonsense variant that could explain or add to the previously identified genetic signal. Therefore, the regulatory potential of rs12041331 region in the PEAR1 locus is still open for investigation. 

The *PEAR1* gene structure includes an enhancer region, where rs12041331 is located, preceded by a CpG island containing several CTCF binding sites [53], these elements all contributing to regulate *PEAR1* expression in megakaryocytes [53,56]. We here aimed to study the DNA methylation profile of *PEAR1* in endothelial cells in order to compare it with the known profile in megakaryocyte precursors [53]. We narrowed a region in *PEAR1* immediately upstream of rs12041331 that displays a cell-specific methylation pattern and interacts with multiple genes involved in protein synthesis and in cell proliferation that were found using available promoter capture HiC data for blood and endothelial cells.

## 2. Results

### 2.1. PEAR1 Hypermethylation in Megakaryocytes but Not Endothelial Cells

**Figure 1:**
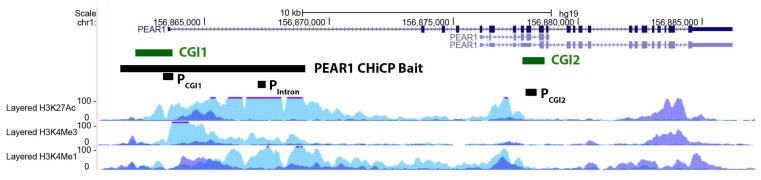
*PEAR1* CHiCP bait overlaps with an enhancer region including *PEAR1* CGI1 and intron 1. Exons are depicted as black boxes, introns as black lines. P_CGI1_, P_intron_ and P_CGI2_ indicate the regions analysed in the methylation study as described in material and methods and [53]. *PEAR1* bait of 7.48 Kb (chr1:156,861,611–156,869,031) identified in the CHiCP analysis is represented as black box. Human Umbilical Vein Endothelial Cells (HUVECs) and K562 H3K4Me1, H3K4Me3 and H3K27Ac profiles are displayed as coloured overlaid histograms (light blue for HUVECs, purple for K562) in “auto-scale to data view” mode that takes the highest signal in the selected region as the 100% of the intensity and display all other signals accordingly (data produced by the Bernstein Lab at the Broad Institute and the UCSC and part of the ENCODE database). *PEAR1* CHiCP bait overlaps with high deposition of the enhancer specific histone marker H3K4Me1 and the promoter specific H3K4Me3. High peaks of the open active chromatin specific histone mark H3K27Ac are also visible in the same region. Adapted from UCSC Browser.

**Figure 2:**
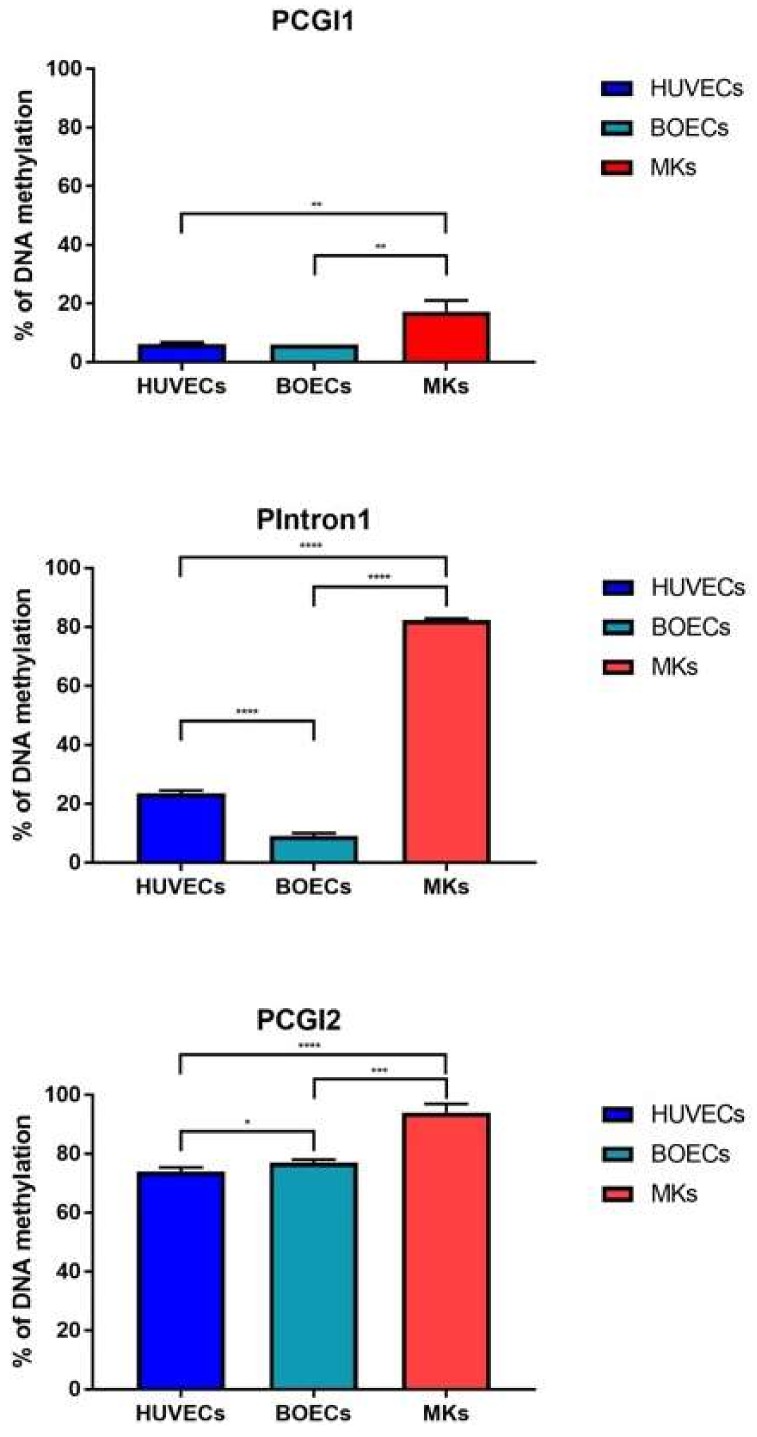
*PEAR1* DNA methylation is higher in megakaryocyte precursors than in HUVECS or Blood Outgrowth Endothelial cells (BOECs). *PEAR1* methylation profile in HUVECs, BOECs and Megakaryocytes (MK) precursors (indicated as “MKs”) analysed on at least 3 biological replicates (data reported as mean +/− SD). * *p* < 0.05, ** *p* < 0.001, *** *p* < 0.0001, **** *p* < 0.00001, unpaired *t*-test.

*PEAR1* methylation was studied in megakaryocytes (MKs) and the endothelial cell lines Human Umbilical Endothelial cell (HUVECs) and Blood Outgrowth Endothelial cells (BOECs) for three different regions of the gene being the CpG Island 1 (P_CGI1_), the intron 1 (P_intron1_) and the CpG Island 2 (P_CGI2_), as previously described [53] (Figure 1). Single CpG methylation values are reported in Supplemental Table S1. MKs showed significant hypermethylation for all the three regions when compared to both HUVECs and BOECs (Figure 2) but the most profound difference in methylation was detected for the intron 1 *PEAR1* region. (Figure 1) This region shows enrichment for histone modifications H3K4Me1 and H3K27Ac, with particular higher deposition in HUVECs (light blue peaks in Figure 1). Active enhancers are in general co-marked by H3K4Me1 and H3K27Ac. [57,58,59].

### 2.2. PEAR1 Associated Long-Distance Chromosomal Interactions in Endothelial Cells and Megakaryocytes

**Figure 3:**
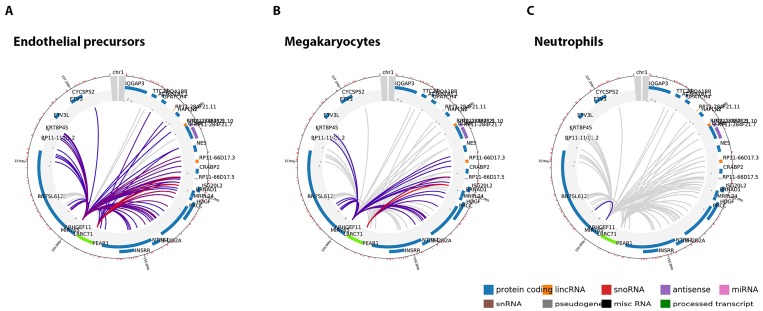
Endothelial precursors and megakaryocytes share similar *PEAR1* interactome. Circular overview of *PEAR1* interactions in endothelial precursors (**A**); megakaryocytes (**B**) and neutrophils (**C**). Two baits are found in *PEAR1* to show interactions, one at the beginning (7.48 Kb, chr1:156,861,611–156,869,031, Assembly 2009, displayed as black box in Figure 1) and one towards the end of the gene (21.26 Kb, chr1:156,881,919–156,903,177, Assembly 2009). Interacting genes are connected by lines and their names are reported on the circle together with their genomic context, defined by colours (protein coding in blue, lincRNA in orange, snoRNA in red, antisense transcript in purple, miRNA in pink, snRNA in brown, pseudogene in grey, miscRNA in black, processed transcripts in green). Interactions with score above 5 are represented with coloured lines with red being the colour referring to the top interactions. Grey lines represent interactions with score below 5, discarded from the analysis.

**Figure 4:**
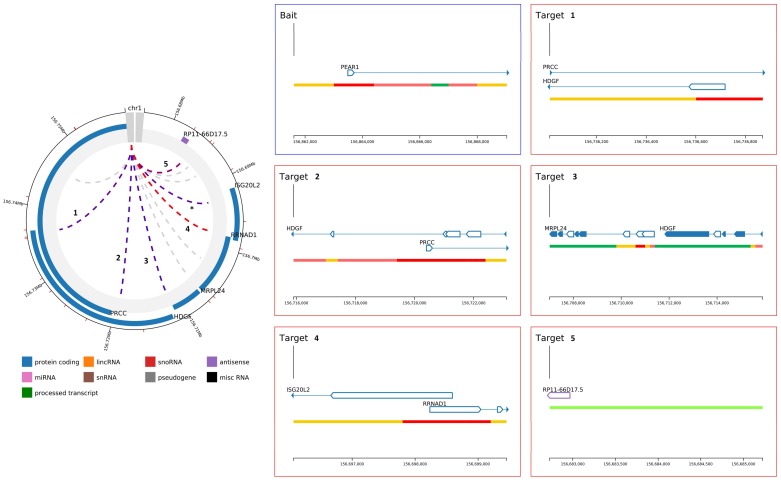
Magnification of *PEAR1* chromosome interactions in endothelial precursors and MKs. Magnification of shared *PEAR1* chromosome interactions among endothelial precursors and MKs in the top left panel. Each interaction is indicated with a number and the correspondent magnification displayed in the right panel reporting gene names, regions of interactions and direction of transcription. *: *PEAR1* interaction that involved a second region of the gene corresponding to location chr1:156,881,919–156,903,177 and not covering *PEAR1* enhancer region analysed in this study.

The methylation region P_intron1_ is located within an enhancer [53] and together with the P_CGI1_ region plays a role in the regulation of *PEAR1* expression. Because of its specific genomic context, we aimed at understanding whether this region is important in chromosomal interactions and this specifically for endothelial cells and megakaryocytes. To study such interactions, we have used available data obtained from promoter capture Hi-C (PCHi-C) experiments that can be visualized using the CHiCP web tool (www.chicp.org) [60] PCHi-C studies reveal the physical interaction between distal DNA regulatory elements and gene promoters at a genome wide scale and these studies have profiled such interactions in several blood cell types [32], including endothelial precursors and megakaryocytes. Therefore, the complete *PEAR1* regulatory region comprising the methylated intron region (chr1:156,863,319–156,863,757, Assembly 2009) was imputed in this database to search for possible specific chromosome interactions. This region partially overlaps with a 7.48 Kb region (chr1:156,861,611–156,869,031) (Figure 1) that was used as bait in the PCHi-C studies that produced the endothelial and megakaryocyte *PEAR1*-specific interactome datasets and is depicted in Figure 3. For both cell types, the *PEAR1* regulatory region is highly connected to the promoter regions of five different genes being *ISG20L2*, *RRNAD1*, *PRCC*, *HDGF* and *MRPL24* and a region that gives rise to a long non-coding RNA with an unknown function (RP11-66D17.5) (Figure 4). Details on these interactions are reported in Table 1. Interestingly, these interactions were not observed in neutrophils (Figure 3C), monocytes or B cells (data not shown). These *PEAR1* interacting genes appeared to be involved in ribosome processing and protein synthesis, cell cycle and proliferation (Table 2) and are mostly identified with Gene Ontology (GO) terms belonging to the transcription regulation pathway. 

**Figure 5:**
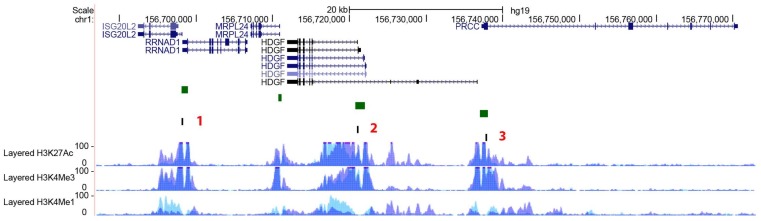
*PEAR1*-interacting genes. Exons are depicted as black boxes, introns as black lines. 1, 2 and 3 in red indicate the regions analysed in the methylation study as described in material and methods and [53] to cover the *ISG20L2/RRNAD1*, *HDGF* and *PRCC* CpG islands (depicted as green boxes). H3K4Me1, H3K4Me3 and H3K27Ac profiles are displayed as coloured overlaid histograms (light blue for HUVECs, purple for K562) in “auto-scale to data view” mode that takes the highest signal in the selected region as the 100% of the intensity and display all other signals accordingly (data produced by the Bernstein Lab at the Broad Institute and the UCSC and part of the ENCODE database). All regions included in the methylation study show high deposition of the enhancer specific histone marker H3K4Me1 and the promoter specific H3K4Me3. High peaks of the open active chromatin specific histone mark H3K27Ac are also visible in the same regions. Adapted from UCSC Browser.

All the *PEAR1* interacting loci, identified in these five genes (Table 1), overlap with CpG islands located approximatively at the beginning of each gene, suggesting that the interaction might depend on and be influenced by the degree of DNA methylation (Figure 4 and Figure 5).

### 2.3. Gene Methylation and Expression Profile of PEAR1-Interacting Genes in Endothelial Cells and Megakaryocytes

To investigate whether DNA methylation at the *PEAR1*-interacting genes could play a role in cell-specific gene regulation, we profiled the methylation status of 3 additional loci located in the promotor CpG islands of *RNNAD1* and *ISG20L2*, *HDGF* and *PRCC* (Figure 5) in both HUVECs and MK precursors. Due to technical reasons depending on the locus characteristics, it was not possible to design an assay to study the methylation of *MRPL24*. Details of the Sequenom assays used are reported in Supplemental Table S2. All these CpG regions remained completely unmethylated in both the cell types (data not shown). In addition to that and contrary to *PEAR1* methylation that significantly changes during megakaryopoiesis using in vitro differentiation assay [53], the CpG island for *RNNAD1*/*ISG20L2*, *HDGF* and *PRCC* remained unmethylated without any changes during MK differentiation.

**Figure 6:**
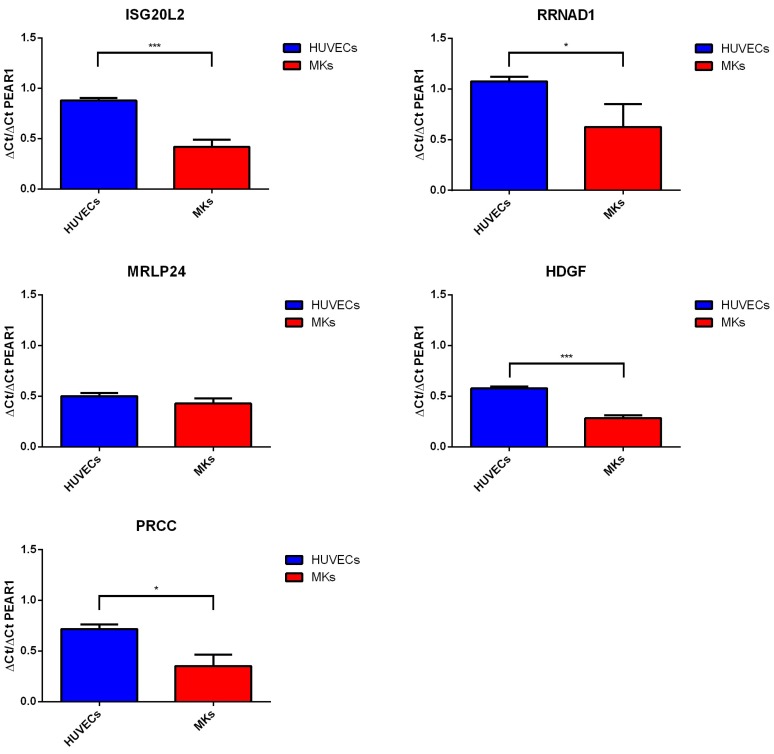
*PEAR1* interacting gene expression. *ISG20L2*, *RRNAD1*, *MRLP24*, *HDGF*, *PRCC* and *PEAR1* expression via quantitative RT-PCR in HUVECs and MK precursors (day 14) from at least 3 biological replicates (mean +/− SD). For each gene data are expressed as “fold” increase relative to GAPDH expression and presented as ratio vs. PEAR1-GAPDH ∆Ct. * *p*-value < 0.05, *** *p*-value < 0.0001, unpaired *t*-test. Details on assays used are reported in Materials and Methods.

To investigate whether *PEAR1* methylation differences in HUVECs and MK precursors could influence distal gene expression through a possible interaction with *PEAR1* CGI1 and the intron 1 region, we studied *PEAR1*, *ISGL20L2*, *RNNAD1*, *MRLP24*, *HDGF* and *PRCC* expression in the same sample sets. Gene fold increase values normalized to GAPDH expression are reported in Supplemental Table S3. *PEAR1* expression was significantly higher in MKs versus HUVECs, following their specific methylation pattern. Therefore, we studied the relative expression of each gene to *PEAR1* expression in both MKs and HUVECs (Figure 6). The ∆Ct/PEAR1-∆Ct ratio for *ISGL20L2*, *RRNAD1, HDGF* and *PRCC* was significantly lower in MKs compared to HUVECs, while no significant difference was observed for *MRLP24* (Figure 6).

## 3. Discussion

We here show that the *PEAR1* regulatory region encompassing both the promoter CpG island (CGI1) and the first intron-enhancer of the gene presents with significantly different methylation profiles when comparing endothelial cells with MKs. Moreover, this region is also highly connected to other genes, as based on chromosomal interaction data for endothelial cells and MKs. 

The *PEAR1* DNA methylation profile in HUVECs, BOECs and MKs mostly differs at CGI1 and intron 1 of the gene. *PEAR1* expression in MKs partially depends upon changes of methylation at CGI1 and high methylation corresponds to high *PEAR1* expression [53]. Our current experiments show that *PEAR1* expression in MKs is higher than in HUVECs, in line with the cell-specific *PEAR1* methylation profiles (Figure 2 and Figure 6).

By studying the methylation of *PEAR1* in HUVECs, BOECs and MKs, we were able to identify a region involved in several enhancer-promoter interactions in endothelial cells and MKs. The same region is also part of a MK specific super enhancer recently identified by Peterson and colleagues in the framework of a genome-wide long-range interactions study [61]. In this study, PEAR1 was found to interact with 7 different genes, 5 of which correspond to our identified PEAR1-interacting loci. Interestingly, three of the characterized *PEAR1* enhancer-interacting genes are involved in important processes related to transcription and protein synthesis (Table 2). *ISG20L2*, *RRNAD1* and *MRPL24* play a role in ribosomal RNA (rRNA) processing and ribosome biogenesis in the cell (*ISG20L2* and *RRNAD1*) [62] or in the mitochondria (*MRLP24*). Gene expression and consequent protein synthesis highly correlate with cell differentiation and proliferation. Several studies have shown that precursors cells contain in general a much higher amount of RNA, normalized to the amount of DNA, compared to specialized cells [63,64]. Transcription profiling studies have revealed that most differentiated cell types express only 10–20% of their genes compared to the 30–60% of embryonic stem cells (ESCs). This pattern is in line with the evidence that cell differentiation moves the chromatin from an open, accessible state up to a more lineage-specific gene expression determined by epigenetic modifications of various types, including DNA methylation [64,65]. In accordance with this evidence, the *PEAR1* interactions with chromosome 1 found, do not involve more mature cell type such as neutrophils (Figure 3). 

Two other chromosome contacts with *PEAR1* involve two genes whose abnormal expression is reported to lead to growth of several tumours [66,67,68,69,70]. *PRCC* is involved in mRNA splicing and reported to have a role in cell cycle delay. *HDGF* encodes for a protein with DNA-binding mitogenic activity involved in cell proliferation and differentiation and is extensively described to be an angiogenic factor in several organs and tumours [71,72,73,74,75]. PEAR1 is known to affect cell proliferation in both megakaryocyte precursors and endothelial cells through the PI3KP3/Akt pathway [43,44] and is involved in neo-angiogenesis [44]. However, so far, no reported data have been shown to link *PEAR1* to cancer development and progression. *PEAR1* interactions with *HDGF* and *PRCC* might modulate their cancer-related role and open up for future research of *PEAR1* in the tumour biology field. In many instances genome-wide disease- and cell trait-associated variants are located in regulatory regions that act distally to influence the expression of other genes [32,35,36,37,38,39,40]. Interestingly, two of the *PEAR1* variants most associated with platelet function and also in linkage disequilibrium with each other, rs12566888 and rs12041331 [45,46,47,48,49,50,51,52,76], are located in the close proximity of the *PEAR1* region involved in chromosome interactions, at 16 and 683 bp of distance from the downstream limit, respectively. Based on our data, future studies should interrogate whether common variants in the *PEAR1* region identified by our analysis, are associated with cancer incidence or progression.

In conclusion, our data suggest that *PEAR1* is not only important as gene encoding for a very well-known contact receptor [42,77,78,79] but might also mediate chromosome interactions with genes involved in protein synthesis, cell proliferation and cancer progression through DNA methylation-dependent mechanisms. 

## 4. Material and Methods

### 4.1. Human HUVECs, BOECs and CD34^+^ Stem Cell Isolation and Differentiation In Vitro

Human Umbilical Vein Endothelial cells (HUVECs), Blood Outgrowth Endothelial cells (BOECs) and Megakaryocytes precursors were isolated from healthy donors and growth in culture as described [43,44,53,80]. HUVECs were freshly isolated from human umbilical veins of healthy volunteers, the day after birth, following a modification of the method of Jaffe et al. [81] Cells were extracted using 0.2% collagenase type 1 (Gibco, Life Technologies, Ghent, Belgium), seeded on gelatine-coated (0.1%) culture dishes in EBM-2 containing EGM-2 BulletKit (Lonza, Walkersville, MD, USA) and cultured (37 °C and 5% CO_2_) until they reached confluence. Formal permission for the isolation of HUVECs was given by the Ethics Committee of the Leuven University Hospitals via isolation of human umbilical cords (Ref. No. ML8663—Approval S54528): informed consent was signed by each mother.

BOECs were isolated from blood of healthy volunteers, as reported previously [44,82]. Briefly, peripheral blood samples were diluted two-fold in PBS and centrifuged on Ficoll Paque (GE Healthcare, 17-440-02, Little Chalfont, UK). Buffy coats were pooled and centrifuged multiple times in PBS. Pellets were resuspended in Endothelial Basal Medium-2 (EBM-2; Lonza) medium and seeded on collagen type-I coated dishes (37 °C, 5% CO_2_) for 30 days (medium was changed daily during the first week and every 2 days thereafter) after which outgrowing colonies were pooled and passaged. After 20–30 days, typical cobble-stone-like colonies appeared and were plated again to reach confluence. BOECs used in this study were harvested at passage 3 after seeding and showed clear endothelial morphology (cobblestone and Weibel-Palade bodies) and phenotype (positive for CD31, VEGR2, CD34, VWF, VE-Cadherin, eNOS). Informed consent was given for the isolation of the blood samples and also this study was granted by the Ethics Committee of the Leuven University Hospitals.

Human CD34^+^ hematopoietic stem cells (HSCs) were separated by magnetic cell sorting from buffy coats isolated from healthy donor peripheral blood (Milteny Biotech, Auburn, CA, USA) and cultured using a protocol described in [53]. The cultured CD34^+^ cells were harvested on days 0, 7 and 14 of differentiation. 

All harvested HUVECs, BOECs and CD34^+^ cells were washed in Dulbecco phosphate-buffered saline (PBS), pelleted and snap-frozen in liquid nitrogen and stored at −80 °C for further DNA and RNA extraction. 

### 4.2. DNA Methylation Analysis

Bisulphite treatment was conducted on 1 μg of genomic DNA using the Methyl Detector kit (Active Motif, Carlsbad, CA, USA) according to the manufacturer’s instructions, except for the incubation protocol during the conversion, performed for a total of 16h as described [83]. Amplicons to study *PEAR1* are already described [53]. Amplicons to study *RNNAD1* and *ISG20L2*, *HDGF* and *PRCC* methylation were designed using the Sequenom (Agena) EpiDesigner software (http://www.epidesigner.com/). Primers and amplicons characteristics are reported in Supplemental Table S2. All PCR amplifications were performed in triplicate. For the CpG- specific analysis, when the triplicate measurements had a SD equal to or greater than 10%, data were discarded. Sequenom (Agena) peaks with reference intensity above 2, overlapping and duplicate units were excluded from the analysis [84,85]. 

### 4.3. CHiCP Analysis

Data on *PEAR1* chromosome interactions in endothelial precursors, megakaryocytes and neutrophils were retrieved from the Capture HiC Plotter (CHiCP, London, UK, www.chicp.org) [60] using data from Javierre et al. [32]. This dataset contains PCHi-C data from 17 blood specific primary cell types (with at least 3 biological replicates) that forms a catalogue of the interactomes of 31,253 annotated promoters. *PEAR1* region chr1:156,863,319–156,863,757 (Assembly 2009) identified in the methylation study was used to search for interaction baits in the database of the endothelial precursors, megakaryocyte and neutrophils interactomes. After identification of the *PEAR1* overlapping bait region present in the CHiCP database, *PEAR1* interactions in endothelial precursors, MKs and neutrophils were considered in the following analysis when their interaction score was above 5. 

### 4.4. Gene Expression Analysis 

Total RNA from HUVECs and MK precursors (day 14) was extracted using TRIzol (Invitrogen, Carlsbad, CA, USA) and cDNA was synthetized following the manufacturer’s instructions (Promega Corporation, Fitchburg, WI, USA). Gene expression for *ISG20L2*, *RRNAD1*, *MRPL24*, *HDGF*, *PRCC* and *PEAR1* was measured using IDT PrimeTime qPCR Primers (Hs.PT.58.39252767, Hs.PT.58.21044077, Hs.PT.58.39042018, Hs.PT.58.97775, Hs.PT.58.21039953 and Hs.PT.58.40290082, respectively) and in-house designed primers for GAPDH (Forward primer: 5′-CTCAGACACCATGGGGAAG-3′ and Reverse primer: 5′-ACGGTGCCATGGAATTTGCC-3′) combined with SYBR green intercalating dye from Life Technologies. Gene expression data were analysed as ratio between each gene’s expression normalized to *GAPDH* and *PEAR1* expression normalized to *GAPDH*.

## Figures and Tables

**Table 1:** *PEAR1* interacting genes.

***PEAR1*** **Bait**	**Endothelial Precursors**	**Location of Target**	**Size Target**	**Name Target**	**Score**	**Genomic Context**
chr1:156,861,611–156,869,031 (7.42 KB)		chr1:156,696,068–156,699,449	3.38 KB	ISG20L2 and RRNAD1	30.34	protein coding
		chr1:156,682,729–156,685,224	2.50 KB	RP11-66D17.5	17.66	antisense
		chr1:156,707,018–156,715,899	8.88 KB	MRPL24 and HDGF	13.62	protein coding
		chr1:156,715,900–156,723,106	7.21 KB	PRCC and HDGF	10.71	protein coding
		chr1:156,736,011–156,736,869	0.86 KB	PRCC and HDGF	10.15	protein coding
		chr1:156,557,122–156,562,660	5.54 KB	APOA1BP and AL365181.1	7.8	protein coding and miRNA
		chr1:156,582,371–156,589,766	7.39 KB	RP11-284F21.11 and HAPLN2	6.65	lincRNA and protein coding
		chr1:156,622,326–156,650,015	27.69 KB	RP11-284F21.7 and BCAN and NES	6.18	antisense and protein coding
	**Megakaryocytes**	**Location of Target**	**Size Target**	**Name Target**	**Score**	**Genomic Context**
		chr1:156,696,068–156,699,449	3.38 KB	ISG20L2 and RRNAD1	22.64	protein coding
		chr1:156,682,729–156,685,224	2.50 KB	RP11-66D17.5	11.97	antisense
		chr1:156,736,011–156,736,869	0.86 KB	PRCC and HDGF	7.86	protein coding
		chr1:156,715,900–156,723,106	7.21 KB	PRCC and HDGF	6.18	protein coding
		chr1:156,707,018–156,715,899	8.88 KB	MRPL24 and HDGF	5.81	protein coding

Nucleotide positions according to the February 2009 human reference sequence (NCBI GRCh37/hg19) produced by the International Human Genome Sequencing Consortium.

**Table 2:** Gene Ontology (GO) terms of *PEAR1* interacting genes.

Gene Name	Protein Name	Gene Ontology (GO)—Biological Processes
***ISG20L2***	Interferon Stimulated Exonuclease Gene 20 Like 2	rRNA processing (**GO:0006364**); ribosome biogenesis (**GO:0042254**); nucleic acid phosphodiester bond hydrolysis (**GO:0090305**); RNA phosphodiester bond hydrolysis, exonucelolytic (**GO:0090503**).
***RRNAD1***	Ribosomal RNA Adenine Dimethylase Domain Containing 1	rRNA methylation (**GO:0031167**); methylation (**GO:0032259**).
***PRCC***	Papillary Renal Cell Carcinoma (Translocation-Associated)	mRNA splicing, via spliceosome (**GO:0000398**); cell cycle (**GO:0007049**); mitotic cell cycle checkpoint (**GO:0007093**).
***HDGF***	Heparin Binding Growth Factor	negative regulation of transcription from RNA polymerase II promoter (**GO:0000122**); transcription, DNA templated (**GO:00006351**); regulation of transcription (**GO:0006355**); signal transduction (**GO:0007165**); cell proliferation (**GO:0008283**).
***MRPL24***	Mitochondrial Ribosomal Protein L24	translation (**GO:0070126**); Mitochondrial translation elongation(**GO:0070125**); mitochondrial translation termination (**GO:0070126**)

## Supplementary Materials

Supplementary materials can be found at http://www.mdpi.com/1422-0067/19/4/1069/s1.
